# (*E*)-3-(4-Bromo-5-methyl­thio­phen-2-yl)acrylo­nitrile

**DOI:** 10.1107/S1600536813019752

**Published:** 2013-08-07

**Authors:** Gamal A. El-Hiti, Keith Smith, Asim A. Balakit, Ali Masmali, Benson M. Kariuki

**Affiliations:** a Department of Optometry, College of Applied Medical Sciences, King Saud University, PO Box 10219, Riyadh 11433, Saudi Arabia; b School of Chemistry, Cardiff University, Main Building, Park Place, Cardiff CF10 3AT, Wales; c Department of Chemistry, College of Science for Women, University of Babylon, Babylon, Iraq

## Abstract

In the title structure, C_8_H_6_BrNS, the molecules are planar with the exception of the methyl H atoms. In the crystal, molecules are linked by intermolecular C—H⋯N interactions to form ribbons parallel to the *b* axis. Groups of ribbons are arranged in a herringbone pattern to form a layered structure parallel to the *ab* plane.

## Related literature

For related structures and their applications, see: Perner *et al.* (2003 ▶); Kose (2004 ▶); Chandra *et al.* (2006 ▶); Zhao *et al.* (2009 ▶); Pu *et al.* (2010 ▶); Dinçalp *et al.* (2011 ▶).
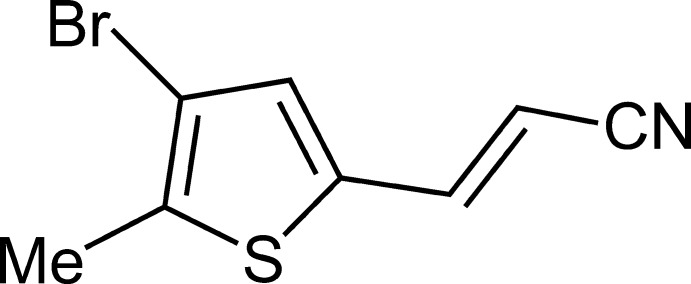


## Experimental

### 

#### Crystal data

C_8_H_6_BrNS
*M* *_r_* = 228.11
Orthorhombic, 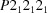

*a* = 6.1347 (5) Å
*b* = 7.1124 (3) Å
*c* = 19.8245 (13) Å
*V* = 864.99 (10) Å^3^
*Z* = 4
Mo *K*α radiation
μ = 4.92 mm^−1^
*T* = 150 K
0.40 × 0.30 × 0.10 mm

#### Data collection

Nonius KappaCCD diffractometer
Absorption correction: empirical (using intensity measurements) (*DENZO*/*SCALEPACK*; Otwinowski & Minor, 1997 ▶) *T*_min_ = 0.243, *T*_max_ = 0.639
3294 measured reflections
1910 independent reflections
1769 reflections with *I* > 2σ(*I*)
*R*_int_ = 0.060

#### Refinement

*R*[*F* ^2^ > 2σ(*F* ^2^)] = 0.048
*wR*(*F* ^2^) = 0.120
*S* = 1.05
1910 reflections
102 parameters
H-atom parameters constrained
Δρ_max_ = 0.74 e Å^−3^
Δρ_min_ = −1.12 e Å^−3^
Absolute structure: Flack (1983 ▶), 699 Friedel pairs
Absolute structure parameter: 0.03 (2)

### 

Data collection: *COLLECT* (Nonius, 2000 ▶); cell refinement: *DENZO* and *SCALEPACK* (Otwinowski & Minor, 1997 ▶); data reduction: *DENZO* and *SCALEPACK*; program(s) used to solve structure: *SHELXS97* (Sheldrick, 2008 ▶); program(s) used to refine structure: *SHELXL97* (Sheldrick, 2008 ▶); molecular graphics: *ORTEP-3 for Windows* (Farrugia, 2012 ▶); software used to prepare material for publication: *WinGX* (Farrugia, 2012 ▶) and *CHEMDRAW Ultra* (Cambridge Soft, 2001 ▶).

## Supplementary Material

Crystal structure: contains datablock(s) I, global. DOI: 10.1107/S1600536813019752/hg5330sup1.cif

Structure factors: contains datablock(s) I. DOI: 10.1107/S1600536813019752/hg5330Isup2.hkl

Supplementary material file. DOI: 10.1107/S1600536813019752/hg5330Isup3.cml

Additional supplementary materials:  crystallographic information; 3D view; checkCIF report

## Figures and Tables

**Table 1:** Hydrogen-bond geometry (Å, °)

*D*—H⋯*A*	*D*—H	H⋯*A*	*D*⋯*A*	*D*—H⋯*A*
C3—H3⋯N1^i^	0.93	2.59	3.501 (8)	166

Symmetry code: (i) 
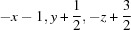
.

**Fig. 1.**
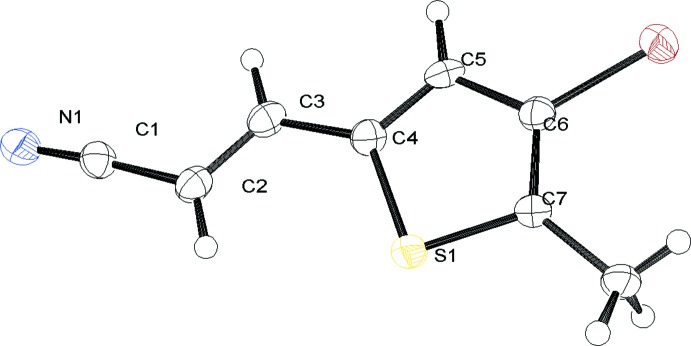
A molecule of I showing atom labels and 50% probability displacement ellipsoids for non-H atoms.

**Fig. 2.**
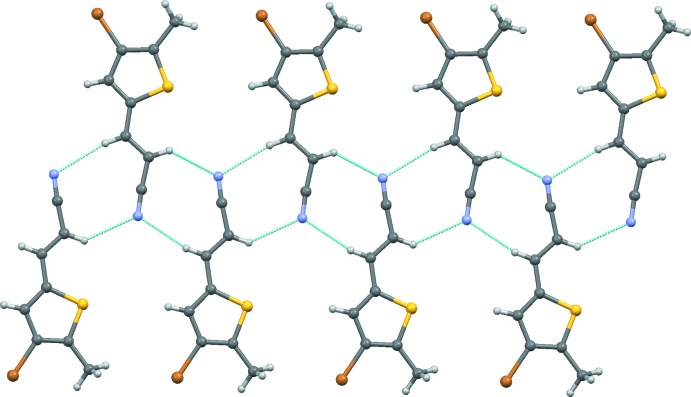
A segment of the crystal structure showing C—H···N interactions as dashed lines.

**Fig. 3.**
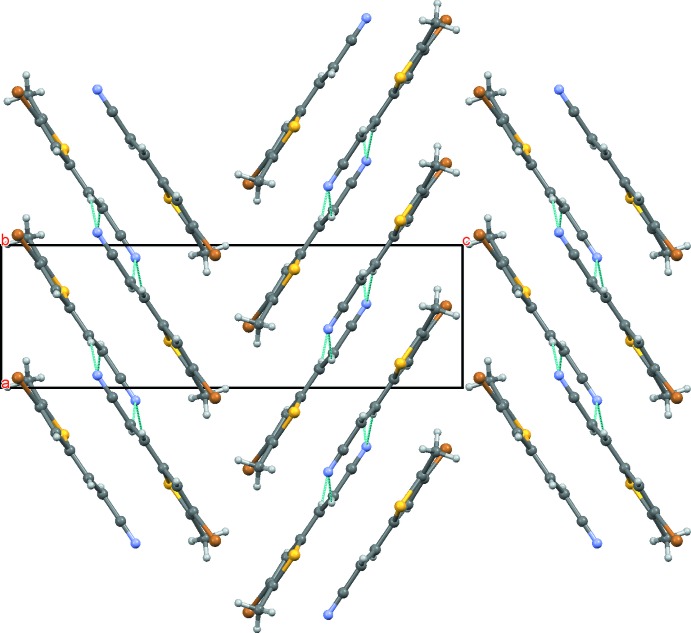
A segment of the crystal structure of I showing the herringbone arrangement to form layers of ribbons.

## supplementary crystallographic information

### 1. Comment

During the research focused on new synthetic routes towards novel substituted thiophene derivatives, we have synthesized the title compound (I), which was isolated in high yield. Thiophene derivatives are interesting compounds (Zhao *et al.*, 2009). They can be used in a wide range of applications such as enzyme inhibitors (Perner *et al.*, 2003), photochromic materials (Kose, 2004; Pu *et al.*, 2010), bioprobes (Chandra *et al.*, 2006) and dyes (Dinçalp *et al.*, 2011).

In the structure, the molecules of (*E*)-3-(4-bromo-5-methylthiophen-2-yl)-acrylonitrile (I) are planar, except for H atoms of the methyl group (Fig. 1). The molecules are linked by C—H···N interactions (Table 1) to form corrugated ribbons. The ribbons run parallel to the *b* axis and, within a ribbon, the orientation of consecutive molecules alternates to the left and right (Fig. 2). Groups of ribbons are arranged in a herringbone pattern to form a layered structure with layers parallel to the *ab* plane (Fig. 3).

### 2. Experimental

Synthesis of*E*-3-(4-bromo-5-methylthiophen-2-yl)acrylonitrile (I)

Diethyl (cyanomethyl)phosphonate (0.94 g, 5.3 mmol) was added to sodium hydride (6.25 mmol) suspended in dry THF (50 ml) under inert atmosphere. The mixture was stirred for 1 h, 3-bromo-2-methylthiophene-5-carboxaldehyde (1.00 g, 4.90 mmol) was added and stirring was continued overnight. Saturated aqueous ammonium chloride solution (25 ml) was added and the mixture was extracted with diethyl ether (4 × 50 ml). The organic phase was washed with saturated aqueous sodium hydrogen carbonate solution (50 ml) and brine (25 ml) and dried over anhydrous magnesium sulfate. The solvent was removed under reduced pressure and the crude product was separated by column chromatography (silica gel, Et_2_O:hexane in 1:1 by volume) to give a mixture of *E*- and *Z*-isomers of 3-(4-bromo-5-methylthiophen-2-yl)acrylonitrile in 4:1 ratio. m.p. 80–81°C. ^1^H NMR (400 MHz, CDCl_3_, δ, p.p.m.): 7.23 (d, *J* = 16.3 Hz, 0.8H), 7.20 (d, *J* = 11.7 Hz, 0.2H), 6.98 (s, 1H), 5.46 (d, *J* = 16.3 Hz, 0.8H), 5.15 (d, *J* = 11.7 Hz, 0.2H), 2.37 (s, 0.6H), 2.35 (s, 2.4H). ^13^C NMR (100 MHz, CDCl_3_, δ, p.p.m.): 141.6 (*d*), 140.0 (*d*), 138.9 (*s*), 135.2 (*s*), 134.8 (*d*), 133.5 (*d*), 117.8 (*s*), 110.9 (*s*), 94.5 (*d*), 91.2 (*d*), 15.4 (*q*). EI–MS (m/*z*, %): 229 ([*M*^81^Br]^+^, 80), 227 ([*M*^79^Br]^+^, 78), 148 (100), 121 (10). HRMS (EI): Calculated for C_8_H_6_BrNS [*M*^79^Br]^+^ 226.9404; found: 226.9402. FT–IR (ν_max_, cm^-1^): 2211. Recrystallization from diethyl ether gave colorless crystals of the *E*-isomer (I).

### 3. Refinement

H atoms were positioned geometrically and refined using a riding model. For sp^2^ H atoms, *U*_iso_(H) is constrained to 1.2 times the *U*_eq_ for the atoms they are bonded to and the C—H distance is 0.93 Å. For the methyl group, *U*_iso_(H) is 1.5 times the *U*_eq_ for C atom they are bonded to and the C—H distance is 0.96 Å, with free rotation about the C—C bond.

### Crystal data

**Table:**

C_8_H_6_BrNS	*F*(000) = 448
*M**_r_* = 228.11	*D*_x_ = 1.752 Mg m^−^^3^
Orthorhombic, *P*2_1_2_1_2_1_	Mo *K*α radiation, λ = 0.71073 Å
Hall symbol: P 2ac 2ab	Cell parameters from 1769 reflections
*a* = 6.1347 (5) Å	θ = 3.0–28.4°
*b* = 7.1124 (3) Å	µ = 4.92 mm^−^^1^
*c* = 19.8245 (13) Å	*T* = 150 K
*V* = 864.99 (10) Å^3^	Plate, yellow
*Z* = 4	0.40 × 0.30 × 0.10 mm

### Data collection

**Table:**

Nonius KappaCCD diffractometer	1910 independent reflections
Radiation source: fine-focus sealed tube	1769 reflections with *I* > 2σ(*I*)
Graphite monochromator	*R*_int_ = 0.060
CCD scans	θ_max_ = 27.4°, θ_min_ = 3.0°
Absorption correction: empirical (using intensity measurements) (*DENZO*/*SCALEPACK*; Otwinowski & Minor, 1997)	*h* = −4→7
*T*_min_ = 0.243, *T*_max_ = 0.639	*k* = −9→7
3294 measured reflections	*l* = −25→20

### Refinement

**Table:**

Refinement on *F*^2^	Hydrogen site location: inferred from neighbouring sites
Least-squares matrix: full	H-atom parameters constrained
*R*[*F*^2^ > 2σ(*F*^2^)] = 0.048	*w* = 1/[σ^2^(*F*_o_^2^) + (0.0412*P*)^2^ + 2.3854*P*] where *P* = (*F*_o_^2^ + 2*F*_c_^2^)/3
*wR*(*F*^2^) = 0.120	(Δ/σ)_max_ < 0.001
*S* = 1.05	Δρ_max_ = 0.74 e Å^−^^3^
1910 reflections	Δρ_min_ = −1.12 e Å^−^^3^
102 parameters	Extinction correction: *SHELXL97* (Sheldrick, 2008), Fc^*^=kFc[1+0.001xFc^2^λ^3^/sin(2θ)]^-1/4^
0 restraints	Extinction coefficient: 0.030 (3)
Primary atom site location: structure-invariant direct methods	Absolute structure: Flack (1983), 699 Friedel pairs
Secondary atom site location: difference Fourier map	Absolute structure parameter: 0.03 (2)

### Special details

**Table:**

Geometry. All e.s.d.'s (except the e.s.d. in the dihedral angle between two l.s. planes) are estimated using the full covariance matrix. The cell e.s.d.'s are taken into account individually in the estimation of e.s.d.'s in distances, angles and torsion angles; correlations between e.s.d.'s in cell parameters are only used when they are defined by crystal symmetry. An approximate (isotropic) treatment of cell e.s.d.'s is used for estimating e.s.d.'s involving l.s. planes.
Refinement. Refinement of *F*^2^ against ALL reflections. The weighted *R*-factor *wR* and goodness of fit *S* are based on *F*^2^, conventional *R*-factors *R* are based on *F*, with *F* set to zero for negative *F*^2^. The threshold expression of *F*^2^ > σ(*F*^2^) is used only for calculating *R*-factors(gt) *etc*. and is not relevant to the choice of reflections for refinement. *R*-factors based on *F*^2^ are statistically about twice as large as those based on *F*, and *R*-factors based on ALL data will be even larger.

### Fractional atomic coordinates and isotropic or equivalent isotropic displacement parameters (Å^2^)

**Table:**

	*x*	*y*	*z*	*U*_iso_*/*U*_eq_	
C1	−0.4336 (10)	−0.1420 (8)	0.7590 (3)	0.0265 (11)	
C2	−0.2429 (10)	−0.1841 (9)	0.7200 (3)	0.0264 (12)	
H2	−0.1959	−0.3079	0.7159	0.032*	
C3	−0.1322 (9)	−0.0462 (8)	0.6895 (3)	0.0258 (12)	
H3	−0.1824	0.0764	0.6946	0.031*	
C4	0.0617 (10)	−0.0763 (7)	0.6489 (3)	0.0235 (11)	
C5	0.1860 (10)	0.0569 (8)	0.6186 (3)	0.0222 (11)	
H5	0.1553	0.1849	0.6203	0.027*	
C6	0.3668 (10)	−0.0199 (8)	0.5843 (3)	0.0241 (12)	
C7	0.3838 (8)	−0.2120 (7)	0.5887 (2)	0.0191 (11)	
C8	0.5528 (11)	−0.3400 (7)	0.5583 (3)	0.0264 (12)	
H8A	0.5059	−0.3791	0.5142	0.040*	
H8B	0.5713	−0.4485	0.5865	0.040*	
H8C	0.6888	−0.2741	0.5546	0.040*	
N1	−0.5883 (9)	−0.1203 (8)	0.7912 (3)	0.0331 (11)	
S1	0.1701 (3)	−0.2987 (2)	0.63524 (7)	0.0246 (3)	
Br1	0.57000 (10)	0.12692 (8)	0.53635 (3)	0.0319 (2)	

### Atomic displacement parameters (Å^2^)

**Table:**

	*U*^11^	*U*^22^	*U*^33^	*U*^12^	*U*^13^	*U*^23^
C1	0.022 (3)	0.028 (3)	0.030 (3)	−0.001 (3)	0.000 (2)	0.000 (2)
C2	0.024 (3)	0.027 (3)	0.029 (3)	0.003 (2)	0.001 (2)	−0.005 (2)
C3	0.023 (3)	0.025 (3)	0.029 (3)	0.006 (2)	−0.002 (2)	−0.003 (2)
C4	0.016 (2)	0.025 (3)	0.030 (3)	0.003 (2)	−0.002 (2)	−0.001 (2)
C5	0.023 (3)	0.018 (2)	0.026 (3)	0.004 (2)	−0.006 (2)	−0.006 (2)
C6	0.025 (3)	0.024 (3)	0.023 (3)	−0.001 (2)	−0.001 (2)	−0.004 (2)
C7	0.021 (3)	0.019 (2)	0.017 (2)	0.002 (2)	−0.0057 (19)	0.0017 (19)
C8	0.032 (3)	0.019 (3)	0.028 (3)	0.002 (2)	0.002 (2)	0.003 (2)
N1	0.031 (3)	0.031 (2)	0.038 (3)	−0.008 (3)	0.002 (2)	−0.004 (2)
S1	0.0240 (7)	0.0207 (6)	0.0291 (7)	0.0007 (6)	0.0021 (6)	−0.0004 (5)
Br1	0.0332 (3)	0.0259 (3)	0.0368 (3)	−0.0021 (3)	0.0065 (3)	0.0040 (2)

### Geometric parameters (Å, º)

**Table:**

C1—N1	1.154 (8)	C5—H5	0.9300
C1—C2	1.434 (8)	C6—C7	1.373 (7)
C2—C3	1.338 (8)	C6—Br1	1.884 (6)
C2—H2	0.9300	C7—C8	1.506 (8)
C3—C4	1.452 (8)	C7—S1	1.717 (5)
C3—H3	0.9300	C8—H8A	0.9600
C4—C5	1.356 (8)	C8—H8B	0.9600
C4—S1	1.737 (5)	C8—H8C	0.9600
C5—C6	1.412 (8)		
			
N1—C1—C2	175.6 (7)	C7—C6—C5	114.5 (5)
C3—C2—C1	120.3 (5)	C7—C6—Br1	122.3 (4)
C3—C2—H2	119.8	C5—C6—Br1	123.3 (4)
C1—C2—H2	119.8	C6—C7—C8	128.9 (5)
C2—C3—C4	123.9 (5)	C6—C7—S1	109.5 (4)
C2—C3—H3	118.0	C8—C7—S1	121.6 (4)
C4—C3—H3	118.0	C7—C8—H8A	109.5
C5—C4—C3	127.1 (5)	C7—C8—H8B	109.5
C5—C4—S1	110.6 (4)	H8A—C8—H8B	109.5
C3—C4—S1	122.3 (4)	C7—C8—H8C	109.5
C4—C5—C6	112.6 (5)	H8A—C8—H8C	109.5
C4—C5—H5	123.7	H8B—C8—H8C	109.5
C6—C5—H5	123.7	C7—S1—C4	92.8 (3)

### Hydrogen-bond geometry (Å, º)

**Table:**

*D*—H···*A*	*D*—H	H···*A*	*D*···*A*	*D*—H···*A*
C3—H3···N1^i^	0.93	2.59	3.501 (8)	166

Symmetry code: (i) −*x*−1, *y*+1/2, −*z*+3/2.
